# Effect of reward downshift on the behaviour and physiology of chickens

**DOI:** 10.1016/j.anbehav.2015.04.005

**Published:** 2015-07

**Authors:** Anna C. Davies, Christine J. Nicol, Andrew N. Radford

**Affiliations:** a Animal Welfare and Behaviour Group, School of Clinical Veterinary Science, University of Bristol, Bristol, U.K.; b School of Biological Sciences, University of Bristol, Bristol, U.K.

**Keywords:** bird, chicken, contrast, expectation, heart rate, latency to reach food, reward, temperature

## Abstract

When a reward is downgraded in quantity or quality from that which is expected, one of two possible outcomes can result. Acquisition responses may decline gradually, owing to a strong stimulus–response reinforcement history, and thus follow the Thorndikian law of effect. Alternatively, there may be an exaggerated reaction to a downgraded reward when it is initially altered, compared to the behaviour of individuals that have always been trained to receive the lower magnitude reward; this is known as successive negative contrast (SNC). While behavioural SNC effects have been commonly demonstrated in mammals, evidence that they occur in other taxa is more equivocal. Additionally, studies demonstrating immediate physiological reactions during reward downshifts are limited. We investigated the reaction of chickens, *Gallus gallus domesticus*, to a downshift in the quality of a food reward that they had been trained to expect in a runway apparatus. During a preshift phase, 16 chickens (control) were given food that was flavoured to make it less preferred, while the other 16 (contrast) were fed the same food but without flavouring. During trial 7, unflavoured food was substituted by flavoured food for contrast hens and all birds were fed the flavoured food during a postshift phase. In the contrast group, food consumption immediately decreased and heart rate increased when the reward was downshifted from unflavoured to flavoured food, but there was no evidence of SNC effects, which could stem from methodological or taxonomic differences from previous studies. The latency to reach the food appeared to follow the Thorndikian law of effect, gradually increasing following the downshift. We suggest that the disparity between the pattern shown by the latency results and other measures could relate to the time period in which measures were taken, as acquisition responses are more likely to follow the law of effect.

## Highlights

• We investigated the reactions of chickens to an unexpected downshift in reward.
• Food consumption decreased and heart rate increased immediately after the downshift.
• Latency to reach food followed the Thorndikian law of effect by increasing gradually.
• There was no evidence of successive negative contrast effects.

A key component of adaptive behaviour in a dynamic world is the formation of expectations, so that decisions about future outcomes can be made rationally (e.g. McCoy & Platt, 2005). A deviation from a normal behavioural reaction might therefore be predicted if expectations are not met. This intriguing hypothesis has led to a large body of research on incentive relativity in both human and nonhuman animals (see review by Flaherty, 1996). Behaviours associated with negative emotions such as ‘regret’, ‘disappointment’ and ‘frustration’ have been well documented in humans when outcomes are poorer than expected (Amsel, 1958; Jensen, Stokes, Paterniti, & Balsam, 2014; Marquis, 1943; Zeelenberg, van Dijk, Manstead, & van der Pligt, 2000). Downshifts in reward magnitude (when a preferred reward is replaced by a less preferred one) have also been shown to result in similar ‘disappointment’ and ‘frustration-like’ reactions in a number of nonhuman species (see reviews by Flaherty, 1996; Papini & Dudley, 1997). However, behavioural reactions to downshift are not always consistent between species (Papini, Mustaca, & Bitterman, 1988).

Two possible outcomes could arise from a reward downshift. The first follows the Thorndikian law of effect, which proposes that the strength of a stimulus–response association is directly related to reward magnitude and probability (Thorndike, 1911). Strong stimulus–response associations therefore make learned behavioural responses more resistant to extinction. This would result in a gradual rather than immediate change, when a preferred reward is substituted by a less preferred alternative. The second, and most widely documented, outcome following a reward downshift is the phenomenon of successive negative contrast (SNC; Flaherty, 1996). SNC effects are characterized by exaggerated reactions shortly after a stimulus is downshifted in magnitude, when compared with control animals that have always been trained to receive the stimulus at the downshifted level (Flaherty, 1996).

An early example of quantitative SNC was observed by Crespi (1942), who studied the speed with which rats, *Rattus norvegicus*, ran down a runway to access a food reward. Compared with control rats that had always been trained to receive a small reward, rats that were shifted from an initial large reward to the same small reward ran more slowly. Crespi described this as a ‘depression effect’ and suggested the analogy to human disappointment. Subsequently, SNC effects have been demonstrated in a range of mammalian species (e.g. Bentosela, Jakovcevic, Elgier, Mustaca, & Papini, 2009; Mustaca, Bentosela, & Papini, 2000; Papini et al., 1988). However, similar experimental procedures have failed to demonstrate SNC effects in most of the nonmammals studied, including turtles, *Chrysemys picta picta* (Papini & Ishida, 1994; Pert & Gonzalez, 1974), goldfish, *Carassius auratus auratus* (Couvillon & Bitterman, 1985; Gonzalez, Ferry, & Powers, 1974; Lowes & Bitterman, 1967; Mackintosh, 1971), toads, *Bufo arenarum* (Schmajuk, Segura, & Ruidiaz, 1981), chickens, *Gallus gallus domesticus* (Petherick, Watson, & Duncan, 1990) and pigeons, *Columba livia* (Papini, 1997; Pellegrini, López, Seal, & Papini, 2008). Instead, these species appear to react to reward downshifts by showing a gradual change in their responses, as predicted by the Thorndikian law of effect.

The species-specific differences in the occurrence of SNC could have an evolutionary basis (Papini, 1997). However, as contrast effects may be an adaptive response to uncertainty (McNamara, Fawcett, & Houston, 2013) and have been demonstrated in one bird, the European starling, *Sturnus vulgaris* (Freidin, Cuello, & Kacelnik, 2009), and one insect, the honeybee, *Apis mellifera* (Couvillon & Bitterman, 1984), methodological differences between studies should be ruled out before taxonomic differences are considered as a potential explanation (Freidin et al., 2009). Apart from Freidin et al.’s (2009) study which used immediately discriminable food types of differing palatability, previous contrast experiments on birds have focused on manipulating reward quantity rather than quality (e.g. Papini, 1997; Petherick et al., 1990). Additionally, previous attempts to obtain evidence for SNC in pigeons and chickens have focused on latency to reach a reward as the sole behavioural measure, without consideration of the effect on behaviour during consumption; changes during consumption were immediately detected in starlings (Freidin et al., 2009). In the current study, we therefore took measures of both latency to reach the reward and food consumption to assess whether previous discrepancies between species are more likely to have a methodological or evolutionary basis.

In addition to behavioural measures, the longer-term effect of reward contrast on physiological processes has been considered to some extent in nonhuman mammals, for example by monitoring corticosteroid level and immune function following a downshift (e.g. Flaherty, Becker, & Pohorecky, 1985; Mitchell & Flaherty, 1998; Pecoraro & Dallman, 2005; Pecoraro, Ginsberg, Akana, & Dallman, 2007; Pecoraro, de Jong, & Dallman, 2009). However, the measurement of more immediate autonomic reactions (e.g. heart rate (HR), skin conductance and blood pressure) at the time of reward downshift is, to our knowledge, largely limited to human studies (see Papini & Dudley, 1997 for review). Generally in humans, an unexpected failure to obtain an expected reward results in increased blood pressure, decreased HR and an initial increase in skin conductance, followed by a decrease. The interpretation of these studies has been challenging, though, owing to the influence of novel rewards and behavioural change (e.g. increased lever pressing; see Otis & Ley, 1993 for an example) on autonomic processes (Papini & Dudley, 1997). In the present study we attempted to dissociate these confounding variables by recording autonomic reaction when the preferred food was downshifted to a less preferred, but not novel, food in a runway task that did not require a behavioural change.

Specifically, we investigated the behavioural and physiological reactions of domestic chickens when an expected reward was altered. We focused on chickens as a study species because their behavioural responses to frustration and anticipation have previously been well studied (e.g. Moe et al., 2009; Zimmerman, Buijs, Bolhuis, & Keeling, 2011; Zimmerman & Koene, 1998; Zimmerman, Koene, & van Hooff, 2000). Additionally, chickens are increasingly being used as a model species for studies of anxiety and depression (e.g. Salmeto et al., 2011), both conditions in which an understanding of anticipation and reward are crucial. During a preshift phase, one group of chickens (contrast) received standard mash and wheat (preferred) food and another group (control) received the standard food with orange oil added as flavouring (less preferred) in a runway apparatus. The standard food reward given to the contrast group was subsequently and unexpectedly substituted with the flavoured food. We measured latency to reach the food throughout the experimental procedure, as monitored in previous studies on birds (Papini, 1997; Petherick et al., 1990). As feeding regulation is sensitive to reward change (Flaherty, 1996; Freidin et al., 2009), we also monitored food consumption throughout the pre- and postshift phases. If chickens react to a reward downshift in accordance with the Thorndikian law of effect, a gradual rather than an immediate change in behavioural acquisition responses would be expected. Alternatively, if chickens react to a downshift with SNC effects, we expected to see immediate behavioural changes, exaggerated in comparison with control values.

The influence of reward contrast on sympathetic nervous system activation was investigated by noninvasively measuring HR and surface body temperature, which might indicate an emotional (frustration-like) response. A decrease in surface body temperature has previously been documented in response to arousing stimuli in chickens (Edgar, Lowe, Paul, & Nicol, 2011; Edgar, Nicol, Pugh, & Paul, 2013; Moe, Stubsjøen, Bohlin, Flø, & Bakken, 2012) and initial decreases in HR occur in humans when an outcome is worse than expected, thought to be a result of attention orienting (Bradley, 2009) or negative feedback processing (Crone et al., 2003). Based on the assumption that the reward alteration would immediately increase autonomic arousal, we predicted that HR would increase and surface body temperature would decrease for the contrast hens compared with control values, when their reward was altered.

## Methods

### Ethical Note

All work was conducted under UK Home Office licence (30/2779). The study also had University ethical approval (UB/12/031) and was conducted in compliance with the ASAB/ABS guidelines. The work involved HR monitoring which occasionally required that a few downy feathers were removed from each hen either side of the keel bone, to allow for skin surface electrode placement. When conducting this procedure, we closely monitored birds for signs of distress (none of which were shown). HR monitoring also required that hens wore a harness containing the monitor. Hen behaviour was assessed throughout habituation for any signs of distress or disruption at wearing the harness. If such distress was observed individuals were not forced to wear the harness. Three hens showed mild distress during the early stages of habituation, so their HR was not monitored for the rest of the study. As we carefully monitored hen behaviour during habituation we do not believe the harness influenced behaviour during testing. Additionally, the study involved short periods (up to 1.5 h) of food deprivation. During these periods, hens were provided with water, housed with other groupmates and monitored for signs of distress (none of which were shown). The hens were housed for 7 weeks during the study period, after which they were rehomed to small responsible freerange holdings.

### Animals, Housing and Husbandry

Thirty-two Hy-line laying hens were obtained at 20 weeks of age from a commercial pullet rearer. Hens were group housed in two different rooms of equal size (3.05 × 3.66 m), each housing 16 experimental birds. An additional room was used as a holding room for habituation and food deprivation of the other birds. Each room contained a two-tiered bank of 10 nestboxes (individual nestbox dimensions: 0.26 × 0.35 m and 0.36 m high), three two-tiered perches (each length of perch was 0.85 m), and were bedded with approximately 10 cm of wood shavings. Ad libitum feed (Farmgate Layers Mash, BOCM Pauls, Ipswich, Suffolk, U.K.) was provided in each room via a large suspended feeder (0.4 m diameter) with 16 individual compartments, and water was available via a hanging drinker (0.38 m diameter). The room temperature was kept at 18–22 °C and the lighting schedule was 12:12 h L:D (light period 0700–1900 hours).

### Experimental and Holding Rooms

The experimental room was separated from the other rooms by a corridor and a solid metal door, to prevent hens from hearing the noise of conspecifics while in the test apparatus. The room contained two wooden pens (1 × 1 m) which were connected by a Perspex tunnel (1.54 × 0.24 m and 0.47 m high; as in Davies, Radford, & Nicol, 2014; Davies, Nicol, Persson, & Radford, 2014). A wooden start box (0.38 × 0.39 m and 0.47 m high) was attached to the centre of the tunnel to form a runway between the start box and the right-hand pen. The pen contained a food bowl which was located on the left-hand side of the pen, 40 cm from the tunnel entrance. A video camera (Kodak Play Sport) was positioned above the start box and another was placed in the pen. A thermal video camera (FLIR SC305) was positioned to record, through a 10 cm diameter hole in the side of the pen, the surface body temperature of hens while they fed from the bowl during their 2 min period within the pen. A set of weighing scales at the back of the room was used to record food consumption during each test.

During habituation and testing, an additional (holding) room was used for short-term (up to 1.5 h) food deprivation. The room was identical to the home rooms in size, but was divided into four sections so that unfamiliar birds from different home rooms did not need to be temporarily housed together. During food deprivation, a metal bell drinker (25 cm diameter) was provided.

### Food Preference Determination

Prior to starting the experiment, we conducted a small study with an additional four hens to identify a preferred and a less preferred food that were identical in appearance and were of the same consistency. The preferred (standard) food consisted of two parts layers mash, combined with one part whole wheat and mixed with five parts water to form a wet feed. The less preferred (flavoured) food consisted of the same composition of wet feed with the addition of orange oil (1 drop/10 g wet food); orange oil has previously been shown to reduce the preference of chickens for a standard diet, although it is not highly aversive (Dixon, Green, & Nicol, 2006; Jones, 1987). During six tests on each individual hen, in which both foods were presented simultaneously, considerably more of the standard food (overall mean (calculated from mean consumption/hen during six trials) ± SE: 10.1 ± 2.1 g) than the flavoured food (0.7 ± 0.4 g) was consumed on each occasion; the sample size of four hens precludes statistical testing.

### Habituation to Handling, HR Monitor, Runway and Food

Hens were first habituated to human presence and handling (days 1–3), then to moving through the runway in groups (days 3–6) and finally to moving individually (days 6–23), until all hens walked through the tunnel without stopping, hesitating or making distress vocalizations. Habituation to HR monitoring was conducted at the same time as runway habituation and began in groups in a separate room (days 5–15) before continuing individually (days 16–23; as in Davies, Radford, et al., 2014; Davies, Nicol, et al., 2014). Habituation to the food bowl was also conducted in parallel with runway trials, with birds fed both the flavoured and standard food in their home groups to ensure the two types were equally familiar (days 14–23).

On day 14, hens were divided into two groups (to give an equal mean body weight in each group) in preparation for testing. During subsequent runway habituation, birds were fed either the flavoured (control group) or standard (contrast group) food from a bowl. Following each trial in the runway, hens were returned to the holding room and were fed (in groups of four) the other food type (flavoured food to the contrast group; standard food to the control group) for 2 min to ensure an equal number of experiences of both. During this period, food consumption was monitored both within the runway test and afterwards in the holding room, to ensure hens maintained a preference for the standard food (which they did). Throughout the habituation phase, various criteria needed to be satisfied (e.g. that hens behaved normally without showing any signs of distress such as escape attempts, freezing or alarm calling) at each stage before individuals could progress (see Davies, Nicol, et al., 2014; Davies, Radford, et al., 2014). Habituation took approximately 4 weeks, depending on individual progression, but there was no significant difference between the mean duration of the habituation periods of control and contrast hens (independent samples *t* test: *t*_30_ = 0.44, *P* = 0.67).

### Test Procedure

Testing consisted of two phases: the preshift phase (contrast group fed standard food, control group fed flavoured food in the runway pen) and the postshift phase (all hens fed flavoured food in the runway pen). Prior to starting the test phase the hens had become well habituated to the test procedure during the habituation phase. The test procedure was identical in each phase. At the start of each trial, a hen was removed from the holding room, taken to the experimental room and the HR monitor was attached. The trial commenced when the HR monitor and a stopwatch were activated simultaneously and the hen was placed in the start box for 10 s. The right-hand wooden panel of the start box was then removed, allowing the hen to see into the apparatus (viewing period), and the tunnel door was removed after a further 10 s, allowing the hen to enter first the runway (runway period) and then the pen (reward period). At the end of the 2 min reward period within the pen, the hen was returned to the holding room. To ensure hens continued to have an equal number of experiences of each food type, after every eight birds (four control, four contrast) had been tested on each occasion, they were fed the alternative food type (from plastic containers) in groups of four for 2 min in the holding room.

Throughout the test period, each hen completed one runway trial twice a day (one in the morning and one in the afternoon) to standardize hunger motivation. All hens were food deprived for 1.5 h in the holding room prior to each trial. Throughout the experiment, hens were tested in the same order, alternating between control and contrast birds. During the preshift phase (trials 1–6), the bowl was filled with 100 g of either flavoured (control group) or standard (contrast group) food (26 g layers mash, 12 g whole wheat, 62 g water). In trial 7, the contrast group was shifted from standard to flavoured food; this feeding regime was retained throughout the postshift phase (trials 8–14).

### Behavioural and Physiological Measures

The latency to reach the food bowl and food consumption were recorded during all trials. Owing to time constraints, physiological measures were recorded only during the morning trials. ECG was monitored as in Davies, Radford, et al. (2014) using noninvasive remote telemetric units (Lowe, Abeyesinghe, Demmers, Wathes, & McKeegan, 2007) and cables contained within a harness. The monitor communicated with a base unit (attached to a computer via USB) and was controlled using RVC Telemetry Software version 1.5 (RVC, Herts, U.K., www.rvc.ac.uk). Measures of HR were extracted using Spike 2 Software version 6 (Cambridge Electronic Design, Cambridge, U.K., www.ced.co.uk) from two 10 s periods: viewing and the first 10 s of the reward period. The percentage change in HR between the viewing and reward periods was subsequently calculated for analyses. Three hens failed to habituate to wearing the harness (two control and one contrast hen), so HR data were not collected for these individuals, reducing the control group sample size to 14 hens and the contrast group to 15 hens. Eye temperature data were extracted from the thermal video using FLIR ResearchIR Software version 1.2 SP2 (FLIR Systems Inc., Wilsonville, OR, U.S.A., www.flir.com), by analysing one clear image of the whole head during the last 30 s of the 2 min reward period. Occasionally, technical difficulties resulted in missing HR or thermal data. When these occurred, missing data points were substituted with a mean value taken from the relevant group (control or contrast) for that trial, to prevent entire rows of data being excluded from the analyses.

### Statistical Analyses

Data were analysed in IBM SPSS Statistics 21 (Armonk, NY, U.S.A.) using repeated measures ANOVAs with two independent groups (control and contrast). For behavioural measures, we initially analysed data from all trials during both the pre- and postshift phases, in line with previous work (Freidin et al., 2009). In the analyses of latency to reach the food, trial 7 was included in the preshift phase (as hens had not experienced the downshift when latency was recorded in that trial). For the food consumption analyses, data collected during trial 7 (when the shift occurred) were included in the postshift phase. Additionally, we conducted analyses on the trials immediately before and after the downshift. We therefore compared trials 7 and 8 when considering latency to reach the food and trials 6 and 7 when considering food consumption.

For the physiological measures, we focused on data collected before and immediately after the downshift to assess any immediate change following reward alteration. This meant comparing trials 5 and 7 (because data were only available from alternate trials).

For each data set, the assumptions of parametric testing were checked using Kolmogorov–Smirnov and Shapiro–Wilk tests. Latency to reach the food data were right skewed and were transformed using a reciprocal transformation. Repeated measures ANOVAs were conducted to consider the effect of treatment (between-subjects effect) and trial number (within-subjects effect). Treatment*trial interaction effects were tested for all response variables, but are presented in the Results only when significant. For analyses considering just the two trials either side of the shift in food type, planned contrasts were subsequently conducted when the interaction term in the initial repeated measures ANOVA was significant; post hoc tests did not therefore employ an adjusted alpha level. Effect sizes are given alongside significant results.

## Results

### Latency to Reach Food

**Figure 1:**
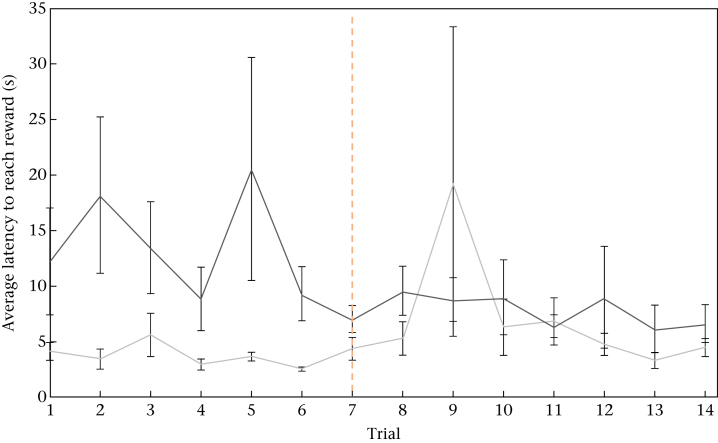
Mean ± 1 SE latency to reach the food across preshift and postshift trials in control (dark grey) and contrast (light grey) groups. The dashed line indicates the shift in food type. *N* = 32 hens.

Hens in the contrast group were significantly quicker to reach the food than those in the control group during the preshift phase (repeated measures ANOVA: *F*_1,29_ = 9.12, *P* = 0.005, eta^2^ = 0.24; trial: *F*_6,24_ = 4.74, *P* = 0.003; Fig. 1). During the postshift phase, there was a strong interaction trend between treatment and trial (*F*_6,25_ = 2.41, *P* = 0.056; trial: *F*_6,25_ = 5.40, *P* = 0.001; treatment: *F*_1,30_ = 2.41, *P* = 0.131): initially, there remained a significant difference between treatments, but this difference became nonsignificant as the postshift phase progressed (Fig. 1). During trial 9 the mean latency for the contrast group was influenced by an outlier, but the same qualitative result was obtained when the outlier was removed. The lack of an immediate increase in latency to reach the food by the contrast group, following the downshift in food, was confirmed by comparing just trials 7 and 8 (the first one following the downshift) when there was a significant effect of treatment (*F*_1,30_ = 8.63, *P* = 0.006, eta^2^ = 0.22; trial: *F*_1,30_ = 3.01, *P* = 0.088), but no significant interaction between treatment and trial (*F*_1,30_ = 0.07, *P* = 0.798). Further tests showed that there was a significant difference between groups during both trial 7 (independent-samples *t* test: *t*_30_ = 2.70, *P* = 0.011, eta^2^ = 0.21) and trial 8 (*t*_30_ = 2.75, *P* = 0.010, eta^2^ = 0.21), but no significant change in latency between trials 7 and 8 for either the control (paired-samples *t* test: *t*_15_ = 1.66, *P* = 0.119) or contrast (*t*_15_ = 1.13, *P* = 0.275) groups.

### Food Consumption

**Figure 2:**
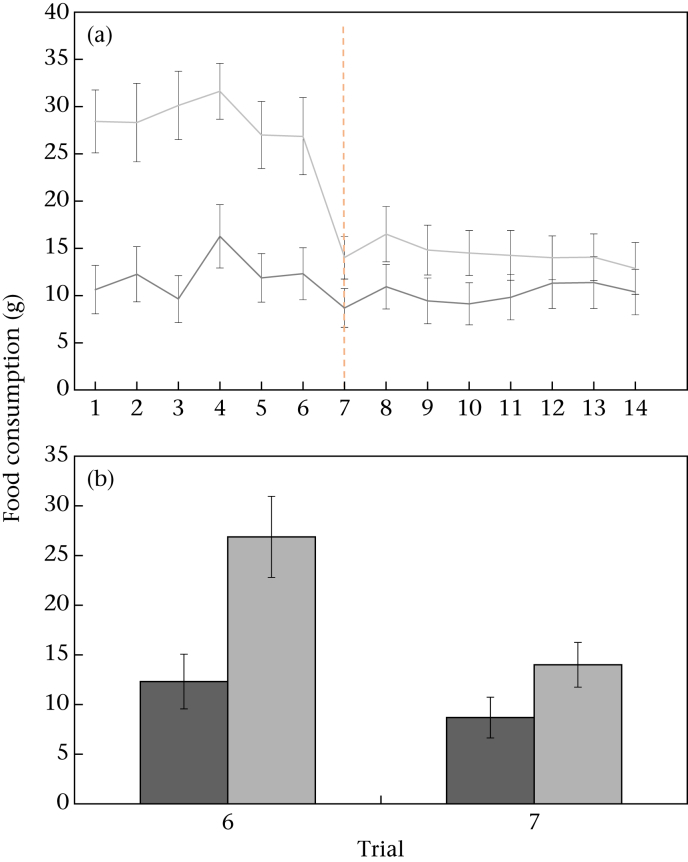
Mean ± 1 SE food consumption (a) across preshift and postshift trials and (b) in trials 6 and 7 in control (dark grey) and contrast (light grey) groups. The dashed line indicates the trial in which the food shift occurred. *N* = 32 hens.

The contrast group consumed significantly more food than the control group during the preshift phase (repeated measures ANOVA: *F*_1,30_ = 15.52, *P* < 0.001, eta^2^ = 0.34; trial: *F*_5,26_ = 2.47, *P* = 0.059; Fig. 2a). During the postshift phase, however, there was no significant difference between treatments in the amount of food consumed (*F*_1,30_ = 1.64, *P* = 0.211; trial: *F*_7,24_ = 1.48, *P* = 0.222; Fig. 2a). When we compared the amount of food consumed during just trials 6 and 7 (when the downshift occurred), there was a significant interaction between treatment and trial (*F*_1,30_ = 9.35, *P* = 0.005, eta^2^ = 0.24; trial: *F*_1,30_ = 29.73, *P* < 0.001; treatment: *F*_1,30_ = 6.82, *P* = 0.014; Fig. 2a). Post hoc tests revealed that there was a significant difference between groups during trial 6 (independent-samples *t* test: *t*_30_ = 2.96, *P* = 0.006, eta^2^ = 0.24; Fig. 2b), but no significant difference during trial 7 (*t*_30_ = 1.75, *P* = 0.091). This change was driven by a significant decrease in food consumption by contrast birds between trials 6 and 7 (paired-samples *t* test: *t*_15_ = 4.91, *P* < 0.001, eta^2^ = 0.63; Fig. 2b).

### Physiological Responses

**Figure 3:**
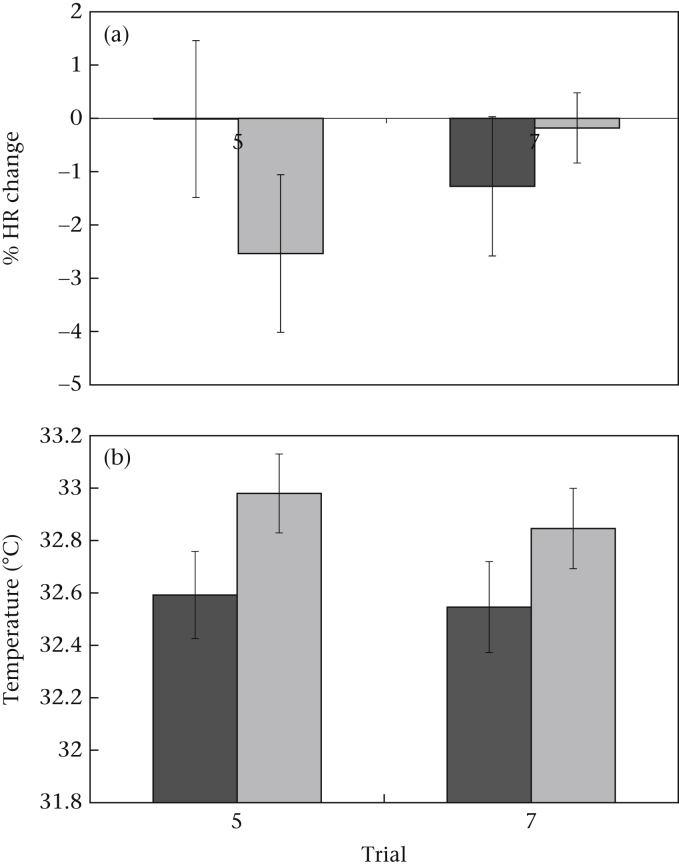
Mean ± 1 SE (a) percentage change in HR between the viewing and reward periods (*N* = 29 hens) and (b) eye temperature during the final 30 s at the food (*N* = 32 hens), during trials 5 and 7 in control (dark grey) and contrast (light grey) groups.

When we considered the difference in HR change (between viewing and reward period) during trials 5 and 7, we found a strong interaction trend between treatment and trial (repeated measures ANOVA: *F*_1,27_ = 4.15, *P* = 0.051; trial: *F*_1,27_ = 0.38, *P* = 0.543; treatment: *F*_1,27_ = 0.21, *P* = 0.650; Fig. 3a). Post hoc tests revealed that there was no significant difference in HR change between trials 5 and 7 for the control group (paired-samples *t* test: *t*_13_ = 0.90, *P* = 0.387), with HR showing a 0% change between the viewing and reward periods in trial 5 and a 1% decrease in trial 7. In the contrast group, however, HR decreased by approximately 2.5% between the viewing and reward period in trial 5 and by 0.2% in trial 7. The extent of the HR decrease was therefore significantly different when the two trials were compared statistically (*t*_14_ = 2.14, *P* = 0.050, eta^2^ = 0.25).

There was no significant effect of treatment on eye temperature in the final 30 s of the reward period during trials 5 and 7 (repeated measures ANOVA: *F*_1,30_ = 2.90, *P* = 0.099; trial: *F*_1,30_ = 0.72, *P* = 0.402; Fig. 3b). The eye temperature also showed no significant change between trials 5 and 7 in either the control (paired-samples *t* test: *t*_15_ = 0.32, *P* = 0.753) or contrast (*t*_15_ = 0.86, *P* = 0.403) groups.

## Discussion

Hens that experienced a downshift in food reward took longer to reach the food than before the shift, but their postshift latency did not differ from that of control birds that had experienced the less preferred food throughout. The change in latency occurred over a few trials, and was thus in line with the Thorndikian law of effect (1911), which predicts a more gradual change in behaviour if there has been a strong reinforcement history, and matches similar response patterns shown in other experiments which have also shown a gradual decline in behavioural responses (Papini, 1997; Petherick et al., 1990). Following reward alteration, there was also a significant decrease in the food consumption of contrast birds, which reached but did not undershoot control group values. In contrast to the change in latency, this was an immediate effect seen in the trial in which the downshift occurred. An immediate decrease in food consumption was also seen in starlings, but it undershot control values indicating SNC (Freidin et al., 2009). Our food consumption result suggests that the chickens learned about, and were responding to the change in, reward properties (a prerequisite for SNC effects) rather than the behaviour simply being strongly associated, but there was no other evidence of an SNC effect.

The disparity between our latency and food consumption results might be a reflection of the period in which each of the measures was taken. Latency to reach the food, which has previously been the only measure in most similar bird studies (Papini, 1997; Petherick et al., 1990, but see Freidin et al., 2009), reflects an expectation of the reward outcome and might induce approach–avoidance conflict (Amsel, 1992). As expectation formation requires either conditioned learning (Tolman, 1932) or a mental representation of future events (Arieti, 1947), it will take a period of time or substantial contrary information (Wilson, Lisle, Kraft, & Wetzel, 1989) for expectations to be adjusted should they not be confirmed. For example, both Petherick et al. (1990) and Papini (1997) found that there was a delay following a downshift in reward magnitude, before latency to reach the food was adjusted. Measures of consumption, however, are taken at the time an individual evaluates the reward and are therefore likely to be more sensitive to reward alteration (Flaherty, 1996). Additional studies on other bird species, taking measures of both food consumption and latency to reach the food, would help to identify whether this is the case.

To our knowledge, the only other study in which measures of food consumption have been monitored in birds was that by Freidin et al. (2009). In that study, it was found that starlings displayed SNC effects. Before considering evolutionary explanations for the interspecific difference in behavioural response, we should therefore consider methodological differences between the two studies. The main difference is in the type of food that was provided during reward alteration. Freidin et al. substituted mealworms for a turkey crumb reward, foods that had different nutritional and physical properties. By contrast, we substituted standard with flavoured food, rewards that were identical in appearance and nutritional content. Although our birds were able to discriminate between the two foods (both latency to reach the food and food consumption were significantly different between treatments in the preshift phase), the similar properties might have meant the alteration was not so salient and resulted in a lack of SNC effects.

The reduced salience of the reward alteration might have also influenced how chickens responded to a change in their expectation. In studies of human behavioural responses to an altered stimulus, it has been found that people assimilate their response to an altered stimulus to their prior expectation (e.g. Wilson et al., 1989), resulting in a lack of ‘disappointment-like’ effects. It has therefore been suggested that a greater contrast effect is likely to result from a greater difference between rewards (Flaherty, 1982). Additionally, in our current experiment the chickens had prior experience of the altered reward and non-novel rewards are associated with a decreased contrast effect in rats (see Flaherty, 1996). Considering one possible function of SNC is to allow an individual to deal with uncertainty in a dynamic foraging environment (McNamara et al., 2013), it might also be expected that SNC effects would be present only if the substituted reward presents a comparatively poor foraging opportunity, for example turkey crumbs versus mealworms in the starling study of Freidin et al. (2009). As there was no reduction in foraging opportunity or in nutritional quality in our experiment, this might also account for the lack of SNC effects. Future studies might beneficially compare responses in the same species to reward alterations that differ in their nutritional value inequality.

In addition to monitoring behavioural responses to reward alteration, we took physiological indicators of sympathetic nervous system activation prior to and immediately after the reward downshift. To our knowledge, this is the first study to take such physiological measures to assess the ‘emotional’ reaction to reward alteration in nonhuman animals. In humans, responses to emotional stimuli are characterized by activation of the parasympathetic branch of the autonomic nervous system which results in an initial deceleration in HR (thought to reflect attentional orienting; see Graham & Clifton, 1966; Bradley, 2009), followed by activation of the sympathetic branch of the autonomic nervous system, which ultimately results in an acceleration in HR (emotional arousal). Hence, interpreting physiological responses in similar human studies has been extremely challenging (Papini & Dudley, 1997). Our study design removed some of the confounding influences on physiology. For example, we substituted the food with an alternative that was less preferred but was not novel (i.e. contrast birds had experienced the flavoured food during habituation) and our task did not require individuals to adjust their behavioural response (e.g. by increasing lever pressing). This reduced the methodological confounds allowing us to determine whether there was any evidence to suggest that the downshift induced an emotional response. The reward alteration had no significant impact on the eye temperature. There was, however, a significant difference in the magnitude of HR change (between the viewing and reward periods) in the contrast group between trials 5 and 7, but there was no significant difference between trials for the control group. The results partly support our prediction that HR would be elevated on discovery of a downshifted reward because the HR of contrast birds was higher in trial 7 than trial 5. In trial 7, however, there was no significant difference between control and contrast HR change, suggesting that the HR did not overshoot control values.

The lack of evidence for physiological SNC effects in chickens may relate to the emotional capacity of the species. Discrete neural circuits identified using electrical brain stimulation and other methods reveal that mammals possess at least seven primary emotional systems, described by Panksepp (2005) as Seeking, Fear, Rage, Play, Lust, Care, Panic. The study of bird emotion is in its infancy and the extent to which avian emotional systems map onto those of mammals is not yet clear. Chickens possess a Seeking emotional system, and show behavioural and physiological signs of frustration (e.g. Zimmerman & Koene, 1998; Zimmerman et al., 2000) when resources are difficult or impossible to access, and anticipation when the arrival of a reward or punishment is expected (e.g. Moe et al., 2009; Moe, Stubsjøen, Bohlin, Flø, & Bakken, 2012; Zimmerman et al., 2011). The apparent lack of SNC effects in chickens may relate to a difference in their emotional systems from that of mammals and may suggest that when developing expectancies, chickens do not use their emotions in the same way as mammals.

To summarize, we found that the food consumption and HR of contrast hens were immediately affected when the reward was downshifted compared with control hens, but there was no evidence of SNC effects. The latency to reach the food appeared to follow the law of effect, by gradually decreasing during the postshift phase. The disparity between the response pattern shown by the latency results and other measures could be due to the time period in which measures were taken. We suggest that further work be conducted in birds to identify whether species-specific or methodological differences could explain the variation in response to a reward downshift.
